# Collective Protection Measures for Occupational Exposure to Carcinogenic Chemicals in France: The Links between Regulations on Chemicals and Effective Implementation

**DOI:** 10.3390/ijerph19148553

**Published:** 2022-07-13

**Authors:** Nathalie Havet, Alexis Penot

**Affiliations:** 1 Laboratoire de Sciences Actuarielle et Financière (LSAF), Institut de Science Financière et d’Assurances (ISFA), Université Claude Bernard Lyon 1, 69007 Lyon, France; 2 Laboratoire de Sciences Actuarielle et Financière (LSAF), Université Claude Bernard Lyon 1, 69007 Lyon, France; alexis.penot@univ-lyon1.fr

**Keywords:** occupational exposure, protection measures, regulations, carcinogens, health inequalities, logistic models, European Union

## Abstract

European directives stipulate that French employers take all available measures to reduce the use of carcinogenic agents. Our study explores the links between regulations on chemicals and the effective implementation of collective protection measures in France to occupational exposure to carcinogenic chemicals. Individual data from the French national cross-sectional survey of occupational hazards, conducted in 2017, were analysed. We investigated whether stricter regulations and longer exposures were associated with a higher level of collective protection using multivariate logistic regressions. In 2017, any collective protection measures were implemented for 35% of occupational situations involving exposure to a carcinogen. A total of 21% of exposure situations benefited from source-based controls (e.g., isolation chamber and local exhaust ventilation) and 26% from general ventilation, for which the effect is limited as collective protection. Our regressions showed that longer exposure durations were associated with more collective protection. Exposure situations to chemicals classified as proven carcinogens by the European Union (category 1A) benefited more from collective protections, which is not the case for products only classified as suspected carcinogens (category 1B). Exposures to products with a Binding Occupational Exposure Limit Value benefited more from source-based controls. Nonetheless, the time spent on the IARC list of carcinogens did not appear to influence the implementation of collective protection measures, except for local exhaust ventilation. At a time when efforts to improve the implementation of protective measures in order to drastically reduce the risks of occupational cancers are still necessary, stricter European and national regulations, but above all, better coordination with the work of the IARC and its classification, are avenues to pursue.

## 1. Introduction

Although significant advances have been made in the fight against cancer, this disease remains a key public health concern and a tremendous burden on European societies. Almost one-quarter of all global cancer cases occur in Europe, which is home to only one-tenth of the world’s population [1,2]. Cancer is the second largest cause of death in Europe after cardiovascular disease: 1.2 million people died of cancer in 2017 [3]. In economic terms, cancer cost the EU almost EUR 97 billion in 2018 [4]. One can still today observe a large number of carcinogenic agents across various occupational settings, while occupational exposures to certain chemical, physical, biological agents and occupational circumstances are well established risk factors for the development of cancer [5,6]. Consequently, approximately 3–14% and 0–2% of all new cancer cases among men and women, respectively, are attributable to occupational exposures in high-income countries [7,8,9,10,11]. In France, around 7900 new cancer cases in 2015 had an occupational origin, representing 2.3% of all new cancer cases (3.9% and 0.4% among men and women, respectively) [12]. In addition to cancers, workplace exposure to carcinogens can lead to other serious health problems, such as respiratory diseases and neurological disorders. The World Health Organization has, therefore, urged governments and industry parties for several years to ensure that workplaces are equipped with adequate means to meet the recommended health and safety standards.

From a regulatory perspective in France, national protection and prevention policies are governed by the European Commission Directives, which are transposed into the French labor code (FLC). Workers are currently protected against cancer-causing substances under three main EU Directives. The overarching *Occupational Safety and Health Framework Directive* (Directive 89/391/EEC) lays out the main principles of workers’ safety and health at work, while the *Chemical Agents Directives* (Directive 98/24/EC) and the *Carcinogens and Mutagens Directive* (CMD) (Directive 2004/37/EC) deal specifically with chemical risks. In particular, the CMD stipulates that employers in the member countries must identify and assess exposure-associated risks for workers, and where risks occur, they must take all available measures to replace carcinogenic agents with a non-hazardous or less hazardous agent or process. Where elimination or substitution is not technically feasible, other measures should be used to lower the exposure level as much as possible. By default, collective protection (e.g., isolation chambers, local exhaust ventilations, physical enclosures, mechanizations of specific procedures) must be implemented, and even preferentially over personal protective equipment (Articles L.230-1 to L.230-5 and R.231-54, FLC) in order to avoid all contact with carcinogenic agents by cutaneous or by respiratory routes. Moreover, employers are also obliged to ensure that occupational exposure limit values for certain carcinogens, established by the CMD or only at the national level for others (Articles R.231-588; R.4412, FLC), are not exceeded.

Given this context, our study examines the disparities that exist in the implementation of collective protection to carcinogenic agents in France. Based on the 2017 French national cross-sectional survey data of occupational hazards, we investigated the influence of the European Union regulations and of the International Agency for Research on Cancer (IARC) classification of carcinogens, in particular, whether stricter regulations are associated with more protection. We also examined whether the most exposed workers benefited from the strongest implementation of collective protection measures. Addressing these issues will allow a better understanding of the legislative drivers in the fight against occupational cancers and whether there is still ample scope for further reductions of the risks through better targeted prevention policies.

## 2. Materials and Methods

### 2.1. Study Population

The SUMER survey (Surveillance Médicale des Expositions aux Risques Professionnels—Medical Monitoring of Exposure to Occupational Risks) is a national cross-sectional survey [13,14,15] periodically conducted by the French Ministry of Labor and the French Directorate for Research, Studies and Statistics to assess occupational risks among a representative sample of the French employee population. Ethics approval was granted by the French National Commission for Data Protection and Privacy (CNIL n°7 62430V1, 2009, updated in 2017) and National Council on Statistical Information. The survey has obtained the label of general interest and statistical quality (visa n°2016X711TV).

The 2017 SUMER survey was based on two-level sampling involving 1243 volunteer occupational physicians, who, over a period of 3 months, randomly selected 33,600 employees for whom they provide medical surveillance in their workplace. A periodical medical examination is compulsory for all employees in France. Full-time occupational physicians were asked to undertake 30 interviews, and the number of interviews was calculated pro-rata for physicians working part-time, with a minimum of 20 questionnaires. A total of 26,500 workers agreed to participate (response rate: 76%) [15].

The physicians assessed individual exposures to 92 different chemical and biological agents over a period of 1 week, based on statements provided by the employees and on their knowledge of the field and the nature of the job or the position. In case of doubt, they could perform a more in-depth workplace assessment. The collected exposure data allowed the selection of 25 chemicals classified as being carcinogenic or probably carcinogenic to humans by the IARC (Groups 1 and 2A, respectively) or classified as known or presumed human carcinogens by the European Union regulations (categories 1A and 1B of the CLP classification). A list of these 25 selected chemicals and their characteristics is shown in Table 1. A total of 2746 workers (10.4%) were exposed to one or more carcinogen at their workplace. Owing to multiple types of exposures for some workers, this corresponded to 4196 identified exposure situations (i.e., the exposure of a worker to a particular entity from the list of the 25 carcinogenic agents).

### 2.2. Collective Protections Variables

For each of these exposure situations, the physician made an assessment of the duration of exposure (reported as a categorical variable), as well as the existing collective protection made available to the employee. In particular, the physician had to report one, and only one, of the following six different scenarios for collective protection for each exposure: (i) ‘no collective protection’; (ii) ‘general ventilation’, which dilutes the pollutant by adding a certain amount of fresh air into the working area; (iii) ‘local exhaust ventilation’, which consists of channelling the flow of pollutants into a ventilation or exhaust system, thereby avoiding their release into the atmosphere of the workplace; (iv) ‘isolation chamber’, allowing for maximum containment of the products or processes, thereby avoiding any contact between the users and the products involved. Work in an isolation chamber requires that all steps of the procedure (e.g., transfer, transport of the product (s), and cleaning and maintenance) abide by this complete confinement. This can result in the mechanisation of the process, adaptation or automatization of specific tasks; (v) ‘other collective protection’; and (vi) ‘availability of collective protection not specified’.

### 2.3. Statistical Methods

Descriptive statistics were used to examine the implementation of collective protections in situations of exposure to carcinogens and the potential differences in regard to the products and the associated regulation (Table 1 and Table 2). In order to test the hypothesis that implementation of collective protection depends on existing regulations regarding their use and substitutability in the workplace, we enriched the SUMER database with variables of regulation for carcinogens:

- One dummy variable indicating whether the agent was classified as category 1A (i.e., a known carcinogen) by the European Union and category 1 by the IARC.
- Two dummy variables indicating whether the agent was classified category 1B (i.e., a suspected carcinogen) by the EU and category 1 (respectively, category 2) by the IARC.
- Two dummy variables indicating whether the agent was classified as a carcinogen solely by the IARC by separating categories 1 and 2.
- A variable was coded ‘1’ if a binding occupational exposure limit value (occupational exposure limit values that take account of socio-economic and technical feasibility factors) (Articles R. 231-58, R. 4412, FCL) was in place for carcinogenic agent before 2017 and ‘0’ otherwise.
- A variable was coded ‘1’ if the carcinogenic agent was substitutable, based on its principal use, as specified by the French National Institute for Research, Occupational Safety and Health (www.inrs.fr (accessed on 1 June 2022)), and the French Agency for Food, Environmental and Occupational Health & Safety (www.substitution-cmr.fr (accessed on 1 June 2022)).
- We also used monographs of the IARC to find the date each agent was entered on the IARC list and created a variable indicating the number of years since the agent was added to the IARC list.

After the descriptive statistics, three separate multivariate logistic regressions were performed, from the broadest analysis to the most precise, by considering successively the following dependent variables: (i) the first, which combined the different types of protection measures, was equal to 1 when ‘a collective protection’ was available and 0 otherwise; (ii) a dichotomous variable was equal to 1 if, for a given exposure situation, there was a ‘source-based control’ (i.e., local exhaust ventilation, an isolation chamber, or other collective protection) and 0 if not. The general ventilation system was separated from all of the other collective protection measures because, in theory, general ventilation offers more limited protection against carcinogenic entities, as it does not prevent direct inhalation of carcinogens by employees; (iii) a dichotomous variable that was equal to 1 if there was a local exhaust ventilation and 0 if not. This regression was to gain a better understanding of the availability of an effective protection, since the very low proportion of isolation chambers did not allow for its analysis on its own. Exposure situations for which the physician had not reported information on protective measures were eliminated for the multivariate analyses. In the three regressions, we included, in addition to the regulation and substitution variables, covariates for the employee characteristics (gender, age, and seniority), for job characteristics (employment contract; work hours; work schedules such as shift work (yes/no), regular night work (yes/no), work on Sunday (yes/no); respondent’s occupation (regrouped into five categories using the French classifications of occupation and socio-professional categories (PCS); close to the international Standard Classification of Occupation (ISO)) and main occupational function (regrouped into five categories: production, manufacturing, and construction; installation, repair, and maintenance; engineering, R&D activities; personal care; others), and for company characteristics (the industry sector (agriculture, and industry; construction; services); company size (1–9 employees, 10–49 employees, 50–199 employees, 200–499 employees, 500 employees and more); geographical location (one variable for each French administrative region); presence of trade union representatives (yes/no); presence of a committee for health, safety, and work conditions (CHSCT) (yes/no); intervention of occupational health and safety (OHS) officers in the last 12 months (yes/no)). We also introduced the duration of the exposure to the carcinogenic agent, with a categorical variable (<2 h; 2–10 h; >10 h over the study period of 1 week) to verify whether longer exposure durations were associated with more collective protection. The statistical analyses were performed using STATA, version 16 software (StataCorp, College Station, TX, USA).

## 3. Results

### 3.1. Descriptive Analysis

In 2017, collective protection was lacking in 35.5% of the 4196 identified carcinogen exposure situations. General ventilation was present in 25.5% of cases, followed by local exhaust ventilation (14.5%). Although they are the most effective ways to protect from exposure to carcinogenic chemicals, isolation chambers were very rarely used by companies (2.3%). Unfortunately, the availability of collective protection was not specified in 18% of exposures. Disparities in collective protection for exposure to the 25 carcinogenic agents were observed. The proportion of exposure without any collective protection varied from 17.5% for cobalt and its compounds to 44% for diesel engine exhaust. The availability of local exhaust ventilation varied from 3.5% for exposures to trichloroethylene to 31.8% for formaldehyde. Isolation chambers were most frequently implemented in cases of exposures to bitumens, coal tar, and its derivatives.

The exposures to products for which a BOELV has been established before 2017 tend to more often involve local exhaust ventilation (18% vs. 12%) and less general ventilation (22% vs. 28%). However, we observe mixed results for the exposure situations for which a substitution principle is applicable, with a lower use of local exhaust ventilation (13% vs. 15%), but a higher implementation of isolation chamber (3.5% vs. 1.7%). Similarly, exposures to products classified as category 1A or 1B by the European Union did not systematically appear more likely to be associated with collective protection than exposures to products only classified by the IARC (Table 2). These descriptive results on regulation should be treated with caution because of potential cofactor effects and differences in the availability of information on collective protection (last column of Table 2); hence, the relevance in having performed multivariate regressions. 

### 3.2. Multivariate Analysis Results

Our multivariate regressions on protection measures revealed substantial discrepancies at different levels (Table 3). At the job characteristics level, managers and technicians were more likely to benefit from collective protective measures (in general and source-based controls) in exposure situations, with all other things being equal. For the exposed employees, this was also the case for workers whose main occupational function was production, manufacturing, engineering, and R&D or personal care. In contrast, Sunday work was associated with a higher probability of not receiving any protection, irrespective of the protection measure studied. Shift work was less associated with source-based controls, while it was less frequently associated with a lack of protection. Shift workers more readily had a general ventilation system available to them, which is of limited effectiveness in regard to the risks associated with carcinogenic products. Similarly, the fact that workers with permanent contracts had a lower probability of not having any collective protection available was exclusively due to the higher probability of general ventilation being available because they had an equivalent probability of having source-based controls available than similar workers with other kinds of contracts. We noted, however, no significant difference in the availability of collective protection between full-time and part-time workers and between day and night workers.

At the company level, less collective protection was available to workers in microenterprises (1–9 employees). Exposed employees in companies with 500 or more employees had a higher probability of being protected due to the more frequent availability of general ventilation systems, while exposed employees in companies with 10–499 employees were more likely to receive protection with source-based controls. The presence of trade union representatives and/or of a CHSCT in the company was not significantly related to the implementation of collective protection. By contrast, intervention of OHS officers in the past 12 months was associated with more implemented protection measures, including source-based controls.

At the product level, the exposure duration had a significant positive impact on the implementation of collective protection. There were fewer source-based controls and collective protections overall for exposures of less than 2 h per week. Exposure situations, for which there is no substitution principle, significantly benefit from the higher implementation of local exhaust ventilation, but did not significantly benefit from the other types of collective protection. Similarly, our multivariate regressions also highlight that the links between implementation of collective protection and regulations were not unequivocal, but seemed to differ depending on the protective measures considered. Situations of exposure to carcinogenic products subject to a BOELV in 2017 benefited 1.4 times more from source-based controls, but not from collective protections in the broader sense, nor from local exhaust ventilation. Exposure situations to chemicals classified as proven carcinogens by the European Union (Category 1A, and all classified 1 in the IARC list) benefited more from source-based controls, including local exhaust ventilation, but not from collective protection in the broader sense. However, more collective protection was not significantly observed for products only classified as suspected carcinogens (Category 1B) by the European Union, even if they were classified carcinogens to humans by the IARC (Category 1). Nonetheless, the entry year for the IARC list had a significant and positive influence on the implementation of some collective protections. More precisely, the older the date of entry of a product on the IARC list, the more likely situations of exposure to that product benefited from local exhaust ventilation. However, this positive effect was non-linear, i.e., it became weaker the longer a product was on the IARC list (significant and negative coefficient associated with the square of the variable ‘time spent on the IARC list’). Consequently, the average probability of benefiting from local exhaust ventilation predicted for our sample increased at a decreasing rate up to the time spent on the IARC list of 15 years, before decreasing (inverted U shape).

## 4. Discussion

Our study explored disparities in France in the implementation of collective protections associated with exposure to 25 carcinogenic chemicals using a large national cross-sectional survey. Firstly, as substantial inequalities in the prevalence and the duration of exposure to carcinogenic, mutagenic, and reprotoxic (CMR) chemicals have been highlighted by previous studies [16,17], we examined whether the same inequalities existed for exposure control strategies or whether the longest exposure situations benefited from more effective protective measures. Secondly, we investigated the influence of the European Union regulations and of the IARC classification of carcinogens on the implementation of collective protection. By addressing these two issues with more recent data and focusing only on carcinogens, our study complements and updates previous work by Havet et al. [18,19,20], conducted on old waves of the SUMER survey prior to the implementation of the European Classification, Labelling and Packaging (CLP) regulation for chemicals.

### 4.1. The Most Exposed, the Most Effectively Protected?

Social inequalities in the implementation of protective measures against carcinogens exposure persisted in 2017. Unfortunately, the workers the most exposed to carcinogenic chemicals were not systematically those that were the most effectively protected. For example, exposed blue-collar workers benefited from less collective protection, and in particular from less source-based controls, compared to exposed managers and executives, while their exposure prevalence and durations were the most substantial [16,17]. Similarly, shift workers who were more exposed to CMR in France [16,17] had a lower probability of benefiting from effective collective protection. Shift workers mainly benefited from general ventilation, which is nevertheless less suitable for a reduction in the risks involved than source-based controls. For these workers, prevention efforts should consist in the reallocation of protective resources to isolation chambers and local exhaust ventilations to improve protective efficiency. Workers in microenterprises also cumulatively had higher exposure prevalence [16,17] and a higher probability of not benefiting from any collective protection in cases of exposure. However, no difference in the implementation of source-based controls was apparent between microenterprises and companies with 500 employees or more. These results indicate that the *National Occupational Health Plan* 2009–2014, for which the top priority target was prevention and protection in small companies, probably helped microenterprises to be able to control occupational exposure to carcinogenic agents by investing in real adapted measures and to catch up with the protection delay they had in the early 2000s compared to very large companies [21]. However, in 2017, exposed employees in medium-sized companies (50–199 employees) were the best protected. Moreover, the interventions of OHS officers in companies, encouraged by the Occupational Health Plans, appeared to be relevant drivers of improvements in the implementation of effective collective protection. By contrast, our study confirms the limited role of the CHSCT in implementation of collective protection [20]; any significant difference in protective measures was apparent between companies with a CHSCT and those that do not have this. Its role in the prevention of exposure to carcinogens may be more through active policies to reduce the prevalence of exposure [17], the solicitation of interventions of OHS officers, or the provision of notification in regard to potential chemical hazards.

A high exposure duration (>2 h/week) was positively associated with the implementation of collective protection, in a broad sense as well as in terms of source-based controls. Having implemented collective protection measures for longer-lasting exposure situations, companies complied with the intentions of the European Carcinogens and Mutagens Directive (Directive 2004/37/EC) and the French Labor Code that stipulate that exposures must be reduced to the lowest possible levels—the best collective protection can compensate for longer exposures. However, in their control strategies, companies did not seem to take into account the duration of exposure beyond 2 h per week. Indeed, situations with more 10 h of exposure per week did not benefit from more collective protection than situations with 2–10 h of exposure per week.

### 4.2. Collective Protection and Regulations

Situations of exposure to carcinogenic products for which the use was subject to strict BOELV significantly benefited more from effective collective protection. The restrictive nature of the limit values for occupational exposure, as compared with the indicative levels, appears to be a decisive factor in prevention policies since it promotes implementation of effective protection measures so as not to surpass the imposed limits. Expanding the list of products with a BOELV appears to be a way to have better collective protection measures, given that a validated measurement method is indispensable for evaluating a BOELV. By the way, the main legislative tool currently used by the European Commission to achieve reductions in health risk from carcinogen exposure at work is the BOELV [22]. Indeed, as part of the fight against cancer under *Europe’s Beating Cancer Plan*, the European Commission proposed amendments to the Carcinogens and Mutagens Directive (CMD) several times, suggesting the expansion of its scope and the inclusion and/or revision of occupational exposures limit values for a number of cancer- or mutation-causing chemical agents (Directives (EU) 2017/2398 in January 2018; 2019/130 in March 2019; 2019/983 in August 2019; 2022/431 in March 2022). For example, the last amendment adds new occupational exposure limits for acrylonitrile and nickel compounds and lowers the limits for benzene. Yet, the EU requirements are gradually transposed into national legislation. Thus, among the list of the 25 selected carcinogens in our study, 6 (wood dust, crystalline silica, asbestos, lead and its compounds, refractory ceramic fibres, and benzene) had a BOELV in France in 2010, 8 (tetrachloroethylene, chromium and its compounds in addition) in 2017 and 12 (acrylamide, formaldehyde, cadmium and its compounds, trichloroethylene in addition) in 2022.

However, the increasing use of BOELV as a tool in the fight against occupational cancers makes even more necessary to find consensus in the debates about how to set limit values [22]. Therefore, works at the European Commission level are currently in progress to move towards a risk-based methodology for setting limit values under the CMD and to adapt the different limit values’ implementation to take into account exposure to a combination of substances acting by the same mode of action.

Our multivariate regressions also suggest that the more stringent regulations in terms of prevention and control (Articles R.4412-1; R4412-3, FLC; EU Classification, Labelling and Packaging (CLP) regulation) associated to chemicals classified as known to have carcinogenic potential for humans by the European Union (Category 1A) improve the implementation of effective collective protection in companies. Nevertheless, the effect of the CLP regulation seems somewhat limited, since situations of exposure to products classified as category 1B, i.e., only presumed to have carcinogenic potential for humans, did not benefit from more collective protection than situations of exposure to agents classified as carcinogens solely by IARC. Similarly, the classification of carcinogens by IARC, which has no mandatory impact on national or supranational legislations, would have a reduced effect on the implementation of effective collective protection, in the sense that situations of exposure to products classified as proven carcinogens (Category 1) by IARC benefited from more protection if and only if the European Union also recognizes the product as known carcinogens. In fact, it is not the category per se of the product (Categories 1, 2A, 2B) on the IARC list that seems important in the control strategies of companies, but rather its entry on the list. The time spent on the list is not decisive, except for the implementation of local exhaust ventilation. One can assume that inclusion on the list has no immediate effects on the implementation of such protective measures; dissemination of knowledge by the IARC is necessary before companies take note for their prevention policies and changes in production processes. Therefore, the current policy of the European Commission to update and revise the CMD in the light of the most recent scientific and technical evidence (including the IARC classification) in a more rapid and continuous process seems to be a relevant approach in the fight against occupational cancers.

Of the 25 carcinogenic agents included in the study, diesel engine exhaust emissions and mineral oils seemed to be the priority chemicals for implementing higher collective protection measures. Indeed, these two chemicals not only had the highest exposure prevalence rates in 2017, but were among the agents for which exposure situations benefited the least from collective protection. Moreover, they were not subject to any BOELV and were not classified as category 1A by the European Union, even though they were considered as known carcinogens by the IARC. What is encouraging is that in 2019, the CMD’s list of occupational exposure limit values was amended to add diesel engine exhaust emissions and mineral oils used before in internal combustion engines. Similar to every EU member state, France has to implement the changes introduced by this amending directive into their own national law. In this way, it is expected that French employees will benefit from better protection when a BOELV for diesel engine exhaust emissions enters into force in 2023, and that certain occupational cancers, especially lung cancers, can thus be prevented.

### 4.3. Limitations of Our Study

Although the SUMER survey is the French national survey, the most relevant to identify exposures to carcinogenic agents, its use has several limitations. The merging of individual products with different levels of carcinogenicity into family of agents is sometimes inaccurate and may have led to imprecision. For example, bitumens are pooled with coal tars while effective protection measures can differ between these products. Moreover, the occupational physicians (the interviewers) did not evaluate the efficiency of protective measures in the event of exposure, but only their presence or not. However, they could be more or less efficient depending on how they were implemented and used. The amount of ‘availability of collective protection not specified’ (18% of exposure) in the SUMER survey constitutes a source of uncertainty that we just cannot deal with. Finally, our cross-sectional survey provides an overview of the collective protection implemented at a given time and the predominant factors associated. A similar study involving several EU countries would be interesting in order to compare the extent of the links between regulation and effective implementation of protection strategies, as each member state may transpose the EU directives into their national legislation in different ways. Nevertheless, to measure the true causal impact of legislation and European directives, future research will need to use longitudinal data that follow the same companies over a long period of time and accurately monitor the introduction and improvement of implemented protective measures as regulations change.

## 5. Conclusions

Social inequalities in the implementation of protective measures against occupational exposure to carcinogenic agents persist to date in France. However, the workers most exposed are not necessarily those that are the most effectively protected. Consequently, there is still ample scope to minimize occupational disease risks. For example, better targeted control strategies, stricter European and national regulations (e.g., the setting of exposure limits for more agents and the lowering of existing limits; reinforcement of the constraints for products classified as proven or suspected carcinogens for humans), and above all, better coordination with the efforts to improve and disseminate knowledge by the IARC are avenues to pursue to drastically reduce the risk of occupational cancers. By setting BOELVs that are designed to protect workers from the worst working conditions, it is hoped that employers will be encouraged to make ongoing prevention efforts to reduce exposures in the workplace by applying good practice and going beyond meeting the minimum standards required.

## Figures and Tables

**Table 1:** Availability of collective protection against the 4196 exposures to 25 selected carcinogenic agents in the 2017 national cross-sectional survey of occupational hazards (SUMER).

Agents	EU Classification	IARC Group	BOELV	Applicable Substitution	Number of Exposures	Collective Protection					
						No	General Ventilation	Local Exhaust Ventilation	Isolation Chamber	Other	Availability Not Specified
Diesel engine exhaust	nc	1	no	no	1092	44.1%	24.9%	7.0%	0.5%	3.8%	18.9%
Mineral oil	1B	1	no	yes	535	37.6%	32.7%	5.2%	3.0%	2.8%	18.7%
Wood dust	1A	1	yes	no	410	34.9%	14.4%	31.5%	0.5%	2.7%	16.1%
Crystalline silica	nc	1	yes	no	384	39.8%	21.9%	12.5%	2.3%	5.5%	18.0%
Formaldehyde (except resin, glue)	1B	1	no	yes	198	26.3%	24.2%	31.8%	2.0%	3.0%	12.6%
Asbestos	1A	1	yes	no	166	33.7%	18.1%	8.4%	6.0%	8.4%	25.3%
Lead and its compounds	nc	2A	yes	yes	152	32.9%	19.7%	11.8%	2.0%	3.3%	30.3%
Chromium and its compounds (except stainless steel)	1A	1 to 3	yes	no	149	20.8%	40.9%	24.2%	2.0%	0.7%	13.4%
Cytostatics	nc	1 to 3	no	no	134	31.4%	27.6%	11.2%	4.5%	16.2%	9.0%
Halogenated aromatic hydrocarbons and/or aromatic nitro compounds	1B	2B to 3	no	no	124	25.0%	36.3%	21.0%	1.6%	3.2%	12.9%
Refractory ceramic fibres	1B	2B	yes	yes	121	38.0%	29.8%	10.7%	2.5%	4.1%	14.9%
Acrylamide	1B	2A	no	yes	105	30.5%	25.7%	20.0%	3.8%	3.8%	16.1%
Nickel compounds	1A	1	no	no	99	26.3%	30.3%	29.3%	1.0%	1.0%	12.1%
Aromatic amines	1A-1B	1 to 3	no	yes	90	33.3%	32.2%	22.2%	1.1%	3.3%	7.8%
Benzene	1A	1	yes	yes	78	20.5%	20.5%	10.3%	20.5%	6.4%	21.8%
Bitumens, coal tar and coal tar pitches	1A	1	no	yes	70	34.3%	17.1%	7.1%	2.9%	7.1%	31.4%
Cobalt and its compounds	1B	2B	no	no	57	17.5%	28.1%	26.3%	7.0%	0.0%	21.1%
Fume emission from metallurgical and electro-metallurgical processes	nc	1	no	no	49	26.5%	36.7%	10.2%	8.2%	4.0%	14.3%
Phenol-formaldehyde resin, urea-formaldehyde, melamine-formaldehyde	nc	1	no	no	38	34.2%	23.7%	10.5%	7.8%	0.0%	23.7%
Cadmium and cadmium compounds	1B	1	no	yes	35	28.6%	34.3%	17.4%	0.0%	0.0%	20.0%
Trichloroethylene	1B	1	no	yes	29	37.9%	17.2%	3.5%	0.0%	0.0%	41.4%
Rubber fumes	nc	1	no	no	28	21.4%	32.1%	28.6%	0.0%	0.0%	17.9%
Metallic carbide	nc	2A	no	no	26	26.9%	30.8%	11.5%	3.8%	0.0%	26.9%
Tetrachloroethylene	2	2A	yes	yes	17	29.4%	17.7%	23.5%	5.9%	0.0%	23.5%
Arsenic and arsenic compounds	1A	1	no	yes	10	20.0%	20.0%	30.0%	0.0%	10.0%	20.0%

Notes: EU: European Union. IARC: International Agency for Research on Cancer. BOELV: Binding occupational exposure limit values. In our analyses, we considered exposure situations rather than carcinogenic agents as such. Thus, we considered current asbestos exposure as not substitutable, given that to date, these mainly result from asbestos removal activities. Similarly, because a large part of diesel exhaust exposure is related to diesel vehicle maintenance and repair, we considered diesel exhaust as not substitutable.

**Table 2:** Implementation of collective protections and regulations.

Product Categories	No Collective Protection	General Ventilation	Local Exhaust Ventilation	Isolation Chamber	Other	Availability of Collective Protection Not Specified
Total	35.6%	25.6%	14.5%	2.3%	3.9%	18.1%
European Union—IARC classifications						
- EU: 1A and IARC: 1	30.6%	22.3%	22.8%	3.1%	3.8%	17.5.%
- EU: 1B and IARC: 1	34.4%	30.1%	12.3%	2.5%	2.6%	18.1%
- EU: 1B and IARC: 2A/2B	29.4%	30.5%	18.4%	3.2%	3.2%	15.5%
- EU: 2/nc and IARC: 1	41.1%	24.9%	9.6%	1.6%	5.0%	17.9%
- EU: 2/nc and IARC: 2A	31.8%	21.0%	12.8%	2.6%	2.6%	29.2%
BOELV						
- Yes	33.9%	21.6%	18.3%	3.0%	4.1%	19.1%
- No	36.5%	27.7%	12.4%	2.0%	3.8%	17.6%
Substitutability applicable to the context of use						
- Yes	33.3%	27.4%	13.2%	3.5%	3.4%	19.2%
- No	36.8%	24.6%	15.2%	1.7%	4.2%	17.5%

Notes: IARC: International Agency for Research on Cancer. BOELV: Binding occupational exposure limit values.

**Table 3:** Results of multivariate logistic regressions for different measures of collective protection.

	No Collective Protection Available		Source-Based Controls		Local Exhaust Ventilation	
	aOR	(95% CI)	aOR	(95% CI)	aOR	(95% CI)
Gender:						
Men	0.843	(0.62–1.15)	1.233	(0.90–1.68)	1.292	(0.90–1.85)
Women	Ref.	Ref.	Ref.	Ref.	Ref.	Ref.
Age	1.000	(0.99–1.01)	1.000	(0.99–1.01)	1.008	(0.99–1.02)
Job seniority:						
<1 year	Ref.	Ref.	Ref.	Ref.	Ref.	Ref.
1–3 years	0.787	(0.52–1.20)	1.162	(0.72–1.88)	1.201	(0.69–2.08)
4–9 years	0.765	(0.51–1.14)	0.943	(0.59–1.50)	0.872	(0.51–1.49
10 years or more	0.802	(0.53–1.21)	0.967	(0.60–1.56)	0.874	(0.51–1.51)
Employment contract:						
Civil servants	Ref.	Ref.	Ref.	Ref.	Ref.	Ref.
Fixed-term contract	0.599 **	(0.40–0.90)	1.159	(0.75–1.79)	1.454	(0.88–2.41)
Permanent contract	0.418 ***	(0.31–0.56)	0.952	(0.69–1.32)	1.207	(0.81–1.80)
Workers with a specific status	0.775	(0.53–1.13)	0.707	(0.44–1.13)	1.275	(0.74–2.20)
Work hours:						
Full-time	0.914	(0.61–1.36)	1.237	(0.78–1.97)	1.163	(0.66–2.05)
Part-time	Ref.	Ref.	Ref.	Ref.	Ref.	Ref.
Shift work:						
No	Ref.	Ref.	Ref.	Ref.	Ref.	Ref.
Yes	0.818 *	(0.66–1.01)	0.606 ***	(0.47–0.78)	0.603 ***	(0.44–0.82)
Regular night work:						
No	Ref.	Ref.	Ref.	Ref.	Ref.	Ref.
Yes	1.222	(0.94–1.59)	1.202	(0.87–1.66)	1.251	(0.84–1.86)
Work on Sundays:						
No	Ref.	Ref.	Ref.	Ref.	Ref.	Ref.
Yes	1.309 ***	(1.09–1.58)	0.648 ***	(0.52–0.80)	0.566 ***	(0.43–0.73)
Occupations:						
Executives and managers	Ref.	Ref.	Ref.	Ref.	Ref.	Ref.
Technicians and associate professionals	1.226	(0.86–1.74)	0.852	(0.60–1.21)	0.894	(0.60–1.34)
Clerks and services workers	2.637 ***	(1.68–4.13)	0.366 ***	(0.21–0.63)	0.348 ***	(0.17–0.72)
Skilled blue-collar workers	1.528 **	(1.08–2.16)	0.500 ***	(0.35–0.72)	0.598 **	(0.39–0.92)
Unskilled blue-collar workers and agricultural workers	1.697 **	(1.13–2.55)	0.352 ***	(0.23–0.55)	0.311 ***	(0.18–0.53)
Main occupational function:						
Production, manufacturing, and construction	0.644 ***	(0.50–0.84)	1.786 ***	(1.30–2.45)	2.620 ***	(1.75–3.92)
Installation, repair, and maintenance	0.775 **	(0.61–0.98)	1.168	(0.86–1.59)	1.852 ***	(1.25–2.74)
Engineering, research and development (R&D) activities	0.414 ***	(0.27–0.64)	2.796 ***	(1.82–4.30)	5.864 ***	(3.53–9.75)
Personal care	0.479 ***	(0.29–0.80)	1.960 **	(1.14–3.37)	2.057 **	(1.00–4.22)
Others	Ref.	Ref.	Ref.	Ref.	Ref.	Ref.
Economic activity:						
Agriculture, industry	0.385 ***	(0.30–0.50)	2.631 ***	(1.94–3.57)	2.978 ***	(2.06–4.30)
Construction	Ref.	Ref.	Ref.	Ref.	Ref.	Ref.
Services	0.319 ***	(0.24–0.42)	2.354 ***	(1.70–3.26)	3.357 ***	(2.27–4.98)
Company size:						
1–9 employees	Ref.	Ref.	Ref.	Ref.	Ref.	Ref.
10–49 employees	0.741 **	(0.59–0.94)	1.291 *	(0.98–1.71)	1.283	(0.92–1.79)
50–199 employees	0.594 ***	(0.42–0.84)	1.642 **	(1.09–2.46)	1.802 **	(1.12–2.91)
200–499 employees	0.417 ***	(0.27–0.64)	1.903 ***	(1.18–3.08)	1.575	(0.89–2.79)
500 or more employees	0.472 ***	(0.32–0.69)	1.432	(0.92–2.23)	1.650	(0.98–2.79)
Presence of a Committee for Health, Safety, and Work conditions (CHSCT)						
No	Ref.	Ref.	Ref.	Ref.	Ref.	Ref.
Yes	1.057	(0.76–1.48)	0.717	(0.49–1.06)	0.756	(0.48–1.20)
Intervention of occupational health and safety (OHS) officers in the last 12 months:						
No	Ref.	Ref.	Ref.	Ref.	Ref.	Ref.
Yes	0.638 ***	(0.53–0.76)	1.544 ***	(1.25–1.90)	1.740 ***	(1.35–2.24)
Presence of trade union representatives in the company						
No	Ref.	Ref.	Ref.	Ref.	Ref.	Ref.
Yes	1.259	(0.94–1.69)	1.054	(0.75–1.48)	0.900	(0.60–1.34)
Substitutability of agent:						
No	Ref.	Ref.	Ref.	Ref.	Ref.	Ref.
Yes	1.171	(0.87–1.56)	0.914	(0.68–1.23)	0.565 ***	(0.40–0.80)
Product with a BOELV:						
No	Ref.	Ref.	Ref.	Ref.	Ref.	Ref.
Yes	0.969	(0.75–1.25)	1.416 ***	(1.09–1.84)	1.123	(0.83–1.52)
Classifications in the EU legislation and IARC list:						
EU: 1A and IARC: 1	0.707	(0.44–1.13)	1.842 **	(1.10–3.08)	2.231 ***	(1.23–4.04)
EU: 1B and IARC: 1	0.951	(0.57–1.58)	1.069	(0.61–1.86)	1.347	(0.71–2.55)
EU: 1B and IARC: 2A/2B	0.754	(0.45–1.26)	1.207	(0.69–2.12)	1.156	(0.71–2.55)
EU: 2/nc and IARC: 1	1.123	(0.68–1.85)	1.042	(0.60–1.80)	0.705	(0.38–1.31)
EU: 2/nc and IARC: 2A	Ref.	Ref.	Ref.	Ref.	Ref.	Ref.
Time spent on the IARC list of carcinogens	0.977	(0.94–1.01)	1.000	(0.96–1.04)	1.066 **	(1.01–1.12)
Time spent on the IARC list of carcinogens ^2	1.000	(0.99–1.00)	0.999	(0.99–1.00)	0.998 ***	(0.99–0.99)
Exposure duration:						
<2 h	1.449 ***	(1.18–1.77)	0.672 ***	(0.53–0.84)	0.613 ***	(0.47–0.80)
2–10 h	1.118	(0.90–1.38)	0.853	(0.67–1.08)	0.990	(0.75–1.30)
10 or more hours	Ref.	Ref.	Ref.	Ref.	Ref.	Ref.
No of exposure situations	3221		3221		3221	

Notes: * *p* < 0.10, ** *p* < 0.05, *** *p* < 0.01; aOR: adjusted odds ratio, 95% confidence intervals in parenthesis; Ref.: reference category for the categorical data. EU: European Union. IARC: International Agency for Research on Cancer. BOELV: Binding occupational exposure limit values In addition to the variables in the table, the models were adjusted for the geographic location of the company (one dichotomous variable for each of 12 French administrative regions).

## Data Availability

The data presented in this study are available on request from the French Ministry of Labor and the French Directorate for Research, Studies and Statistics for research purposes.
